# Phytochemicals Analysis and Some Bioactive Properties of *Erica manipuliflora* Salisb. (EMS); Antibacterial, Antiradical and Anti-lipid Peroxidation

**DOI:** 10.22037/ijpr.2021.115270.15288

**Published:** 2021

**Authors:** Arzu Kavaz Yüksel, Emrah Dikici, Mehmet Yüksel, Mesut Işik, Fatih Tozoğlu, Ekrem Köksal

**Affiliations:** a *Department of Food Technology, Technical Sciences Vocational School, Atatürk University, Erzurum, Turkey. *; b *Science and Technology Application and Research Center, Aksaray University, Aksaray, Turkey. *; c *Department of Food Engineering, Faculty of Agriculture, Atatürk University, Erzurum, Turkey. *; d *Department of Bioengineering, Faculty of Engineering, Bilecik Şeyh Edebali University, Bilecik, Turkey. *; e *Department of Food Processing, Vocational School, Bilecik Şeyh Edebali University, Bilecik, Turkey. *; f *Department of Chemistry, Faculty of Science and arts, Erzincan Binali Yıldırım University, Erzincan, Turkey.*

**Keywords:** Erica manipuliflora Salisb., Phytochemical analysis, Antioxidant, Acetylcholinesterase, Lipid peroxidation, Antibacterial

## Abstract

In the present study, the phenolic content by using LC MS/MS method, anticholinergic, antioxidant (metal reduction, radical and removal of lipid peroxidation), and antibacterial activities of *Erica manipuliflora *Salisb. (EMS) extract were determined. The amount of vanillic acid, fumaric acid, catechin hydrate, quercetin, and phloridzin dihydrate were found higher than other compounds. The ethanol extract of the EMS showed an inhibition effect on the Acetylcholinesterase (AChE) enzyme with IC_50 _value of 0.124 ± 0.008 mg mL^-1^. Also, this extract of the EMS indicated radical (DPPH and ABTS) scavenging activity (about 20%) and the reducing capacity for Cu(II) and Fe(III) close to trolox, and inhibited the oxidation of linoleic acid with 40.5% at 20 **μg** mL^-1^. Antibacterial activity of the extracts was investigated against *Staphylococcus aureus,* *Escherichia coli*, and *Salmonella *Typhimurium using agar disc diffusion and minimum inhibitory concentration (MIC) methods. The EMS extract was found to be effective when used at least 312 mg mL^-1 ^concentration on the pathogenic microorganisms. Consequently, it has an important non-synthetic natural content that can be used in the treatment of many diseases due to its many bioactivities such as anticholinergic, antioxidant (radical removal, lipid peroxidation prevention, etc.) and antibacterial.

## Introduction

The medicinal plants contain many bioactive and antimicrobial agents as pharmacologically and microbiologically. Significantly, they have been used for different purposes in Turkey and worldwide for many thousands of years. Many of the plants have antioxidant, and antimicrobial activity in living organisms and foods because of their compound contents (1). Therefore, they have some advantages when used by humans. For example, they are natural, less expensive, and have fewer side effects on health and better patient tolerance. The plants contain phenolic compounds are important for the food industry due to their lipid peroxidation reducing capacity and nutritional value improve properties. The interest in using natural antioxidant-based plants increases day by day instead of synthetic antioxidants, because synthetic antioxidants have possible toxicity and carcinogenic effects when used in the food industry (2).

The *Erica *genus belongs to the *Ericaceae* family, which contains 4250 species, spread across 124 genera, and has a large family in the world. There are 12 genera and about 30 species of this family in Turkey, and they are widely found in the coastal regions of Turkey. There are five species of this genus in Turkey* Erica* species, including; *E. manipuliflora, E. arborea, E, bocquetii, E. spiculifolia *salisb. *Erica *species are named “funda”, “süpürge çalısı” or “püren” in different parts of Turkey. *Erica manipuliflora* S., a member of the *Ericaceae *family, and the homeland of this species is the Eastern Mediterranean. It is located in our country’s Aegean and Mediterranean coastal zone, especially under or beside the pine forests (3).

Flavonoids and phenolics are important compounds in plants and fruits. They are the members of the natural antioxidants and have great importance for human health due to the antioxidant, and some other properties. The dried leaves and flowers of *Erica* species, especially *E. arborea* and *E. manipuliflora *are used as a natural drug in Turkish folk medicine. The therapeutic properties of its are generally stemmed from a variety of polyphenolic substances in them (4). The flowers and leaves of the species, which are used in medicine and pharmacy, can be drunk as an infusion, decoction, and fresh herbal tea. When the leaves and flowers of this plant are fresh and prepared with 5% water and taken 2-3 times a day, it might have a diuretic, urinary tract disinfectant, stone-lowering, and constipation removal effect. Also, the obtained drugs from this plant are widely used as antiulcer (5, 6), cytotoxic, antibacterial, antiedema, diuretic, antiseptic agent, antimicrobial, cytoprotective, wound healing activity, antidiarrheal, antiseptic, diuretic, antiviral, astringent, antioxidant, anti-inflammatory, antinociceptive and analgesic (3,7 and 8).

Alzheimer’s disease (AD) is an important illness; a current drug-discovery strategy wants to develop agents with multiple potencies, including inhibition of acetylcholinesterase (AChE). The AChE is located in red blood cells and brains at high concentrations. This enzyme is involved in the hydrolysis of the neurotransmitter acetylcholine. Compounds with inhibitory properties on cholinesterase such as AChE and butyrylcholinesterase (BChE) is important for the treatment of AD. The discovery of potential AChE inhibitors from natural products as an alternative due to the negative side effects of synthetic drugs used to treat AD has attracted significant interest (9).

Spices, medicinal plants, and some vegetables are known primary sources of natural antioxidants. In the food industry, synthetic antioxidants are used as lipid oxidation inhibitors, and synthetic antimicrobials are used to prevent microbial spoilage. Therefore, researches on natural antioxidants and antimicrobials have intensified in recent years. The *Erica* plant includes phytochemicals, especially flavonoids, phenolics, and (-)-epicatechin and quercetin. *Erica* species have certain total polyphenolic compounds, including flavonoids, tannins, anthocyanins, glycosides, terpenes, and some other compounds (10). Lipid oxidation is one of the most important factors for shelf life and protection properties for pharmaceutical and food products. The oxidation process undesirable off-flavors and decreases the nutritional safety and quality of food products, which are unacceptable to consumers (11). Therefore, it is important to know natural products with content such as phenolic or flavonoid that prevent lipid peroxidation and have radical scavenging activity.

The medicinal plants show some properties on pathogen microorganisms, especially inhibition of their growth and reduction in the number of pathogens. It is known that plants contain specific bioactive molecules. These compounds show an inhibition of bacterial or fungal growth in the environment and foods. Also, the antimicrobial compounds can inhibit the pathogens. For that reason, they are considered for the development of some antimicrobial drugs (12). Since the late 19th century, scientific studies have reported some antimicrobial properties of certain herbs, plants, spices, and specific components.

In this study, we aimed to determine the effects of ethanolic extracts of EMS on radical removal and metal reduction activity as well as inhibitory properties on AChE. The phenolic content analysis was determined by LC-MS/MS method, and the enzymatic inhibitory effects were associated with antioxidant activity. The antimicrobial effect and MIC data of this plant extract were determined. Also, this study aimed to evaluate the antibacterial activity of these plant fractions against the *Staphylococcus aureus*, *Escherichia coli*, and Salmonella *Typhimurium.*

## Experimental

*Material*

EMS samples were collected at the flowering period in March-April 2019 from Muğla (Latitude: 37°13′05″ N, Longitude: 28°21′59″ E, Altitude: 672 m) in Turkey. The plants were botanically identified by the Faculty of Pharmacy, Istanbul University (Herbarium code number; ISTE 110340; 110341). The obtained aerial parts (2.50 kg) of plant material from the same area was washed with deionized water and dried for 5 to 7 days in the shade at room temperature, and milled using a grinder (Lavion Herb Spice Grinder Mill Machine) and stored in flasks at -4 °C until analyzed (13). 

*Preparation of the extraction*

The extraction was carried out using the method described previously with minor modifications (14). The dried sample/solvent (ethanol) ratio was mixed in a ratio of 1/10, and the extraction process was carried out in a shaker for 24 h. Then the extracts were filtered using Whatman No.1 filter paper. Then, it was filtrated, the solvent was evaporated and lyophilized. The dry matter was kept in a closed container at +4 °C until analysis. LC-MS/MS analysis results were calculated considering the total dry matter ratio.

*Content analysis by LC-MS/MS method*

Content analysis of EMS was performed by LC-MS/MS, using a Nexera model Shimadzu UHPLC attached to a tandem MS device. The validation studies of the method developed for 20 phenolic substances were carried out in the Harran University Central Research Laboratory. The LC-30AD dual pumps, DGU-20A3R degasser, CTO-10ASVP column furnace, and SIL-30AC autosampler were used to carry out the chromatographic processes. Chromatographic separation was performed on a C18 Intersil ODS-4 (3.0 mm × 100 mm, 2 µM) analytical column. The column temperature was fixed to 40°C. The elution gradient was created from mobile phase A (Water, 0.1% Formic acid) and mobile phase B (Methanol, 0.1% Formic acid). The solvent flow rate was kept at 0.5 mL min^-1^, and the injection volume was set to 4 µL. MS detection was done using a mass spectrometer equipped with Shimadzu LC-MS 8040 model triple, quadrupole, and ESI source operation in both positive and negative ionization modes. LC-MS/MS data calculations were made with Lab Solutions software (Shimadzu, Kyoto, Japan). Multiple reaction tracking (MRM) mode was used to measure (15, 16). In the experiment, three applications were made for each compound analysis, averaged, and the results were presented quantitatively.

**Evaluation of antioxidant activities**

*Total reduction capability*

The reduction power of EMS extracts was performed in a modified form of the method reported by (17). Different amounts of EMS extracts (10, 20, 40 µg mL^-1^) were mixed with 2.5 mL phosphate buffer (0.2 M, pH 6.6) and 2.5 mL 1% potassium ferricyanide [K_3_Fe(CN)_6_]. The mixtures were incubated at 50°C for 20 minutes. After incubation, trichloroacetic acid (2.5 mL, 10%) and FeCl_3_ (0.25 mL, 0.1%) were added to each mixture and centrifuged (at 3,000 rpm for 10 minutes). The absorbance values of the mixture at 700 nm were recorded. The increased absorbance with the reaction in the mixture indicated that its reduction capacity was increased. The reducing power results are expressed as absorbance and compared to standard antioxidants.

*Cu* ^++^ *ion reduction capacity (CUPRAC)*

The CUPRAC method based on the reduction of Cu (II) -Nc to Cu (I) -Nc chelate was applied (18). One mililiter of neocuprin (2,9-dimethyl-1,10-phenanthroline), 1 mL of CuCl_2_ (0.01 M) solution, and 1 mL of ammonium acetate (NH_4_Ac) buffer solution were added to the test tube and mixed with a vortex. Then extracts of different concentrations (10, 20, 40 µL) were added, and the total volume was adjusted to 4 mL with water. After incubation for 30 min at room temperature, the absorbance was recorded at 450 nm. The increased absorbance with the reaction in the mixture indicates that the Cu ion reduction capacity increases. The reducing power results are expressed as absorbance and compared to standard antioxidants. The increased absorbance with the reaction in the mixture indicates that the Cu ion reduction capacity increases.

*DPPH* ^•^ *removal activity*

The DPPH^•^ free radical removal activity of EMS extracts and standard antioxidants was performed by the Blois method (19). The DPPH solution (0.1 mM) was prepared in ethanol. The sample obtained from stock solutions (10, 20, 40 μg mL^-1^) was mixed with 1 mL of this solution and made up to 3 mL with ethanol. These solutions were thoroughly vortexed and incubated for 30 min in the dark. Absorbance was measured with a spectrophotometer at 517 nm. The low absorbance of the reaction mixture indicates that the higher radical removal activity. Results are expressed as percent radical scavenging activity.

*ABTS* ^•+^ *removal activity*

This method is based on the principle of color change resulting from the treatment of colored ABTS^•+^ cation radical with an extract (20). ABTS^•+^ cation radical was mixed with ABTS (2 mmol L^-1^) solution and 2.45 mmol L^-1^ potassium persulfate (K_2_S_2_O_8_) solution. The obtained solution was incubated in the dark and at room temperature for 14 h. Before using the ABTS^•+^ cation radical, ABTS^•+^ radical solution was diluted with sodium phosphate buffer (0.1 mol L^-1^, pH 7.4) until obtained an absorbance as 0.750 ± 0.025 at 734 nm. Then, 10, 20, 40 µL of extracted stock solutions were taken, and phosphate buffer was added until its volume was 3mL. The prepared 1 mL ABTS^•+^ solution was stirred with the extracts and vortexed. Radical removal activity was measured at 734 nm. Results are expressed as percent radical scavenging activity.

**Determination of acetylcholinesterase (AChE) activity**

In this study, the inhibitory effect of different concentrations of EMS extract on the AChE enzyme was investigated. The inhibitory effect of ethanol extract on the AChE enzyme has been tested using the Ellman spectrophotometric method (21). Reaction solution containing 50 µL 5,5′-dithio-bis (2-nitro-benzoic) acid (DTNB), 100 µL Tris – HCl buffer (1 M, pH 8.0) and 50 µL AChE (5.32 × 10^-3^ U) incubated at 30 °C and stirred for 15 min. Finally, the reaction was started with the addition of 50 µL of acetylthiocholine iodide (AChI) used as a substrate. Enzymatic hydrolysis of the substrate was recorded spectrophotometrically at a wavelength of 412 nm, and then the IC_50_ value was determined (22). The effect of EMS ethanol extract in different concentration ranges (0.8-0.25 mg mL-1) against the AChE was screened. The IC_50_ values were calculated from activity (%)-[Ligand] graphs for extract (22, 23).

**Linoleic acid peroxidation removal activity** **by ferric thiocyanate method**

Linoleic acid peroxidation removal activity was determined according to the ferric thiocyanate method (24). This method is based on measuring the hydroperoxide formed by linoleic acid oxidation spectrophotometrically at 500 nm. High absorbance indicates that the excess amount of peroxide formed as a result of peroxidation. The formed hydroperoxide oxidizes Fe^2+^ to Fe^3+^. Then, Fe^3+^ forms a complex with the added thiocyanate, and this complex gives a maximum absorbance at 500 nm. The concentrations corresponding to the desired amounts were transferred from stock solutions (1 mg mL^-1^) to vials by an automatic pipette, and volume was completed with buffer solution (2.5 mL, 0.04 M, pH 7.0). Then 2.5 mL of the linoleic acid emulsion was added to each vial. 2.5 mL buffer solution and 2.5 mL linoleic acid emulsion were used as control. Incubation was carried out at 37 ^o^C. One-hundred microliter from the vials were taken and placed in test tubes containing 4.7 mL of ethanol every six hours. 100 µL Fe^2 + ^solution and then 100 µL SCN^- ^solution were added to them. The blind sample was prepared by adding 100 µL Fe^2+ ^and 100 µL SCN^- ^solutions to the test tube containing 4.8 mL ethanol. Absorbances of the samples at 500 nm were read against the blind sample. The inhibition effect of samples on lipid peroxidation was expressed as percent scavenging activity (25, 26).

**Determination of antibacterial activity**

*Preparation of cultures*

For the determination of the antibacterial effects of extracts, *Escherichia coli* (ATCC 25922), *Staphylococcus aureus* (ATCC 25923), and *Salmonella *Typhimurium (ATCC 14028) cultures were used that stored at -80 ° C. These cultures were activated in Trypticase Soy Yeast Extract (TSYE) during 24 hours at 35-37 °C and their densities were adjusted to 1 × 10^8^ CFU mL^-1^ using the 0.5 McFarland standard.

*Minimum inhibitory concentration (MIC) method*

EMS extracts were prepared in 35% Dimethyl sulfoxide (DMSO) at the concentration of 312 mg mL^-1^ (w/v) and sterilized using 0.45 µm filters. The sterile samples were stored in 1.5 mL Eppendorf tubes at 4 °C until antimicrobial activity tests were performed. The bacterial inoculums (10 µL) was added inoculum was added to the microplates, and the plant extract was diluted to 312, 156, 78, 39, 20, 10 mg mL^-1 ^using nutrient broth (NB). DMSO is used as a negative control. The prepared microplates were incubated at 35-37 °C for 24 h. The visual turbidity samples were accepted as positive, and cultivation was done from them. The turbidity and growth in the plates were interpreted as a positive situations. At the end of the incubation period, the plates were evaluated for the presence or absence of growth. Each test was repeated at least three times (27).

*Disc Diffusion method*

For this analysis, sterile and 9 cm diameter petri dishes were prepared using 20 mL of Mueller-Hinton medium. The standard quantities of each bacterial suspension (10^8^ CFU mL^-1^) were transferred to the prepared mediums. After 5 min, 20 µL of EMS extract impregnated sterile paper discs (6 mm) were placed on the mediums. For the acceleration of the extract diffusion into the agar, the plates were kept at 4 °C for 1 h, and then incubated for 24 to 48 h at 37 °C. Diameters of bactericidal areas (mm), including the control disk, were evaluated. Ciprofloxacin (5 μg disk^-1^) was used as a positive control for this analysis (27).

*Statistical study*

Data analysis was realized using GraphPad Prism version 6 (GraphPad Software, La Jolla, California USA). The results were exhibited as mean ± standard deviation (95% confidence intervals). Differences between data sets were considered statistically significant when the *p*-value was less than 0.05.

## Results and Discussion

*Quantitative determination of phenolic compound content by LC-MS/MS*

Many studies have shown that phenolic compounds have some biological activities such as antioxidant, antimicrobial, and antitumor. In general, it has been reported that the antioxidant activities of phenolic and flavonoids may depend on the chemical structure of the compounds and the distribution of hydroxyl groups. Thus, phenolic compounds can protect cellular components against oxidative damage and therefore reduce the risk of degenerative disease due to oxidative stress (28). In this study, it was reported that the phenolic compounds in the *Erica* herbal extract were effective for preventing lipid peroxidation. Also, *Erica *extracts contain flavonoids and other phenolics that can contribute to total antioxidant activity (10). 

In this study, the phenolic compounds (flavonoids or phenolic acids) composition of EMS was evaluated with the phenolic compounds introduced as standard. Linear regression quotations and linearity ranges of standard compounds available in the instrument library are given in Table 1. In the analytical method used, LOD and LOQ varied in the range of 0.5 μg mL^-1^-206.8 μg L^-1 ^and 0.1 μg mL^-1^-214.3 μg L^-1^, respectively. Recovery of phenolic compounds; ranges from 96.6% to 101.1%. In the literatüre, when the validation studies performed for the determination of phenolic compounds were examined, it was seen that they were in parallel with the data in this study. The R^2^ values are within the limits of the data available in the literature. In a study on method development, the correlation coefficients were higher than 0.999, and the recoveries varied between 95.9% and 106% (29). In the LC-MS/MS validation study performed for some plant species, the LOD/LOQ varied in the range of 0.05 μg mL-25.8 μg L^-1^ and 0.17 μg mL^-1^-85.9 μg L-1, respectively, and the recovery of phenolic compounds ranged from 96.9% to 106.2% (30). These results supported our quantitative phenolic analysis.

As seen in Table 1, it was determined that the amount of vanillic acid (520.01 µg L^-1^), fumaric acid (411.06 µg L^-1^), catechin hydrate (261. 27 µg L^-1^), quercetin (174.36 µg L^-1^), myricetin (131.18 µg L^-1^) and phloridzin dihydrate (98.31 µg L^-1^) were higher than acetohydroxamic acid, caffeic acid, resveratrol, gallic acid and oleuropein, bütein and kaempferol in the range of 84.37µg L^-1^-8.83 µg L^-1^ (Table 1). The compounds we detected in the study are mainly from flavonoid glycosides, phenolic acids, and derivatives. In a study, content analysis of *Erica arborea* L. samples was made using LC-MS analysis, and 72 different phenolic compounds were identified. They have classified all of these components into four different groups: (1) flavonoid aglycones, (2) flavonoid glycosides, (3) phenolic acids and derivatives, and (4) flavan-3-ols and proanthocyanidins (31). As stated in the literature, it can be said that these species show many bioactive properties with their richness in phenolic compounds and, therefore may have therapeutic effects for many diseases. 

Vanillic acid is a phenolic derivative of some plants and fruits. It is also formed as an intermediate product during the production of vanillin from ferulic acid. The earlier studies reported that vanillic acid has a hepatoprotective effect of the acetaminophen (APAP)-induced toxicity in rats (32). Adipic, tartaric, citric, ascorbic, malic, and fumaric acid were employed as antioxidant and antimicrobial properties (33). Catechin hydrate is one of the important flavonoids derived from plants as a secondary metabolite. It has gained attention due to its potential anti-inflammatory and antioxidant properties. On the other hand, its important properties have been found to prevent and treat diseases stemmed from oxidative damage Quercetin and myricetin have some abilities to prevent the oxidation of low-density lipoproteins (LDL) chelating transition metal ions and scavenging free radicals. Generally, it might prevent certain diseases, such as chronic inflammation, atherosclerosis and cancer. Although myricetin occurs in less common foods than quercetin, it is consumed at lower levels than quercetin. Some researchers reported that myricetin could have greater antioxidants activity than quercetin. But, it might be less active than quercetin for the inhibition of low-density lipoproteins (34).

*Antioxidant activities of EMS*

It has been suggested that a wide variety of phenolic content of plants might result from the differences in their structural properties. According to the researchers, the significant correlations between phenolics and antioxidant activity may support the hypothesis that phenolic compounds found in plant extracts can contribute to the total antioxidant capacity (35). The beneficial effects of vegetables, plants, and fruits might be explained by their high antioxidant properties.

DPPH assay test is generally used for the measurement of free radical scavenging activities of antioxidants. This method is stemmed from the reduction of a DPPH solution in alcohol with the source of a hydrogen donating antioxidant. ABTS radical cation is calculated with the spectrophotometric method (36). In a study, the DPPH scavenging effect of *E. manipuliflora* methanol extracts was investigated, and it showed a radical scavenging effect of approximately 50 % at 255 mg mL^-1^ concentrations (37). While 18% radical scavenging activity was shown in the current study, this rate was 50% at 100 times lower concentration in the previous study. In another study, methanol (IC_50_: 0.38. ± 0.002 mg mL^-1^) and hexane extract (IC_50_: 0.20 ± 0.001 mg mL^-1^) of this plant exhibited ABTS radical scavenging activities (3). As seen in Table 2, The EMS ethanol extract showed about 22% ABTS radical scavenging activity and 18% DPPH radical scavenging activity at a concentration of 0.2 mg mL^-1^. In the antioxidant activity test performed according to the DPPH method, the highest antioxidant activity was determined in the standard of torolox (0.2 mg mL^-1^) (measuring 81.19%) and it was followed by BHA (0.2 mg mL^-1^) (measuring 71.82%), BHT (0.2 mg mL^-1^) (46.33%), and EMS (0.2 mg mL^-1^) (measuring 18.31%), respectively. Also, similar findings were determined using the ABTS method. In other words, the antioxidant activity decreased according to this order: Torolox> BHA>BHT>EMS. The antioxidant activities of these plants might be stemmed from the absence of some specificity components. Similar results were reported by Sengul *et al.,* (12) for “*Artemisia absinthium*, *Saponaria officinalis, *and* Artemisia santonicum *extracts“. The multifunctional antioxidant activity of polyphenols is highly correlated with phenol rings (acting as electron traps) that play an active role in scavenging many reactive radical species (38).

The reduction assay can be used to measure the total reducing capacity of plant extracts and antioxidant compounds (39). The CUPRAC assay is a rapid, simple, cost-effective, selective, and steady antioxidant determination method for a wide variety of polyphenols. CUPRAC reactions might be completed approximately in 30 min (18). In a study, extracts of different parts of *E. manipuliflora* were prepared and found to have metal reduction potential (3). As seen in Table 2, the iron and copper reducing activity of EMS is close to trolox, indicating that its antioxidant capacity is high. The highest reduction capability was determined in BHT, and it was followed by BHA, while trolox, and our plant extract showed similar activity. The absorbances value was measured for trolox as 0.516 ± 0.012; while CUPRAC assay value was measured for EMS as 0.401±0.009, respectively. The obtained results showed that the used plant extract had a good copper removal activity. Also, it might be said that phenolic compounds such as vanillic acid, fumaric acid, catechin hydrate, caffeic acid, resveratrol, gallic acid, oleuropein, butein, and kaempferol that found in EMS can contribute significantly to radical removal and metal reduction capacity.

*The anticholinergic effect*

Overactivity of the acetylcholinesterase (AChE) enzyme, which hydrolyzes the neurotransmitter acetylcholine, causes Alzheimer’s disease (AD). AChE inhibitors used in the symptomatic treatment of AD are known to increase antioxidant production and protect cells from oxidative damage (40).

In a study, the inhibition effect of aqueous extracts of E. manipuliflora on AChE was investigated, and it showed an inhibition effect of approximately 50% against AChE enzyme at 200 µg mL^-1^ concentrations (41). They reported a lower anticholinergic effect compared to the current study (124 µg mL^-1^). As shown in Table 2, EMS ethanol extract had an inhibition effect on AChE (IC_50_: 0.124 ± 0.008 mg mL^-1^, R^2^: 0.965). Köroğlu *et al.,* (42) usually found higher values for total phenolic content and antioxidant activity than our findings, while Dias *et al*., (43) reported similar results with our studies. Many studies have reported that flavonoid and phenolic compounds have anti-acetylcholinesterase activity. Because of the neuroprotective effects of phenolic compounds, they may play an important role in treating AD. One of the most important approaches for the treatment of this disease is to raise the acetylcholine rate in the brain with AChE inhibitors. AChE activity is also effective in intestinal health and AChE inhibitors are used to treat gastric disease, abdominal pain, vomiting, and constipation. Also, Dias *et al.,* (43) and Kuş *et al.,* (3) reported that several types of *Ericaceae* family showed a very strong inhibitory activity against acetylcholinesterase (AChE) and butyrylcholinesterase (BChE) enzymes. In this study, considering the LS-MS/MS content analysis, caffeic acid, vanillic acid, silymarin, lutein, resveratrol and other phenolic acids in the content of EMS have been reported to have an inhibitory effect against AChE (2, 44 and 45). In the light of this information in the literature, it can be said that the radical removal potential of EMS ethanol extract may be due to its phenolic compound content.

*Linoleic acid peroxidation removal activity of EMS*

**Figure 1:**
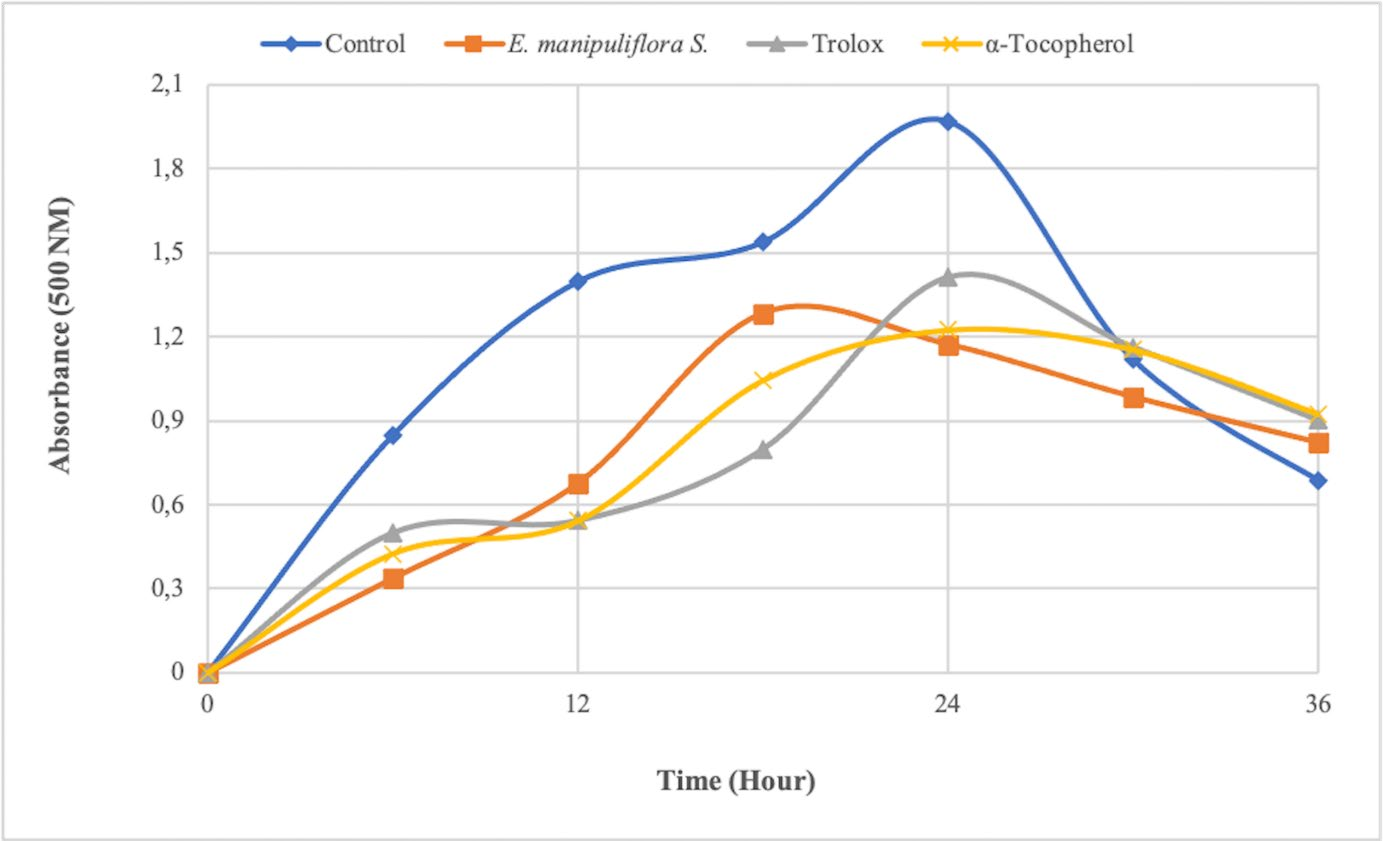
Linoleic acid peroxidation inhibitory activity of standard antioxidants (α-tocopherol and troloks) and EMS extracts (20 µg mL^-1^).

The ability to remove linoleic acid peroxidation by the ferric thiocyanate method is one of the most used antioxidant parameters. This method measures the amount of peroxide formed during lipid peroxidation. In this analysis, hydroperoxides are oxidized by the oxygen of the air and formed because of peroxidation of linoleic acid in the emulsion that is indirectly measured. In a study, the inhibition effect of *E. manipuliflora* extracts (ethyl acetate) on lipid peroxidation was investigated, and it showed an inhibition effect of approximately 50% at 45 µg/mL concentrations (41). They reported a lower lipid peroxidation inhibitory effect compared to the current study (20 µg/mL). In the study, it was found that the sample extract (20 µg mL^-1^) inhibits lipid peroxidation for up to 36 h (Figure 1). The inhibition effect of EMS, trolox, and α-tocopherol on the linoleic acid oxidation was found to be approximately 41%, 28%, and 38%, respectively, at 24^th ^h. The inhibition effect of this plant extract on the peroxidation of the linoleic acid emulsion was higher than the standards. As a result of our study, it can be said that the high lipid peroxidation inhibitory activity may be due to the compounds in the plant content. In a previous study, it has been stated that many phenolic and flavonoid compounds have an inhibitory effect on lipid peroxidation (46). These results suggest that phenolic compounds found in plant content could be a suitable natural antioxidant for preventing the oxidation of fatty acids and foodstuffs containing them.

The obtained results showed that the phenolic compound contents of EMS exhibited significant anticholinergic and antioxidant activities (metal reduction, radical and lipid peroxidation removal). In addition, this research revealed that this plant, which had a naturally rich source of antioxidants, might be used to prevent many diseases such as Alzheimer’s and atherosclerosis.

*Antimicrobial properties of EMS*

The plants that have some medicinal properties are being picked up and used by humans to treat being picked up and used by humans for the treatment of various diseases in the world and our countries for centuries. Although technology and medicine have developed extensively in recent years, humans preferred to use natural products due to their natural and harmless properties on health. In the study, antimicrobial activities of the extract obtained from EMS plant against three different microorganisms, namely *Staphylococcus aureus*, *Esherichia coli* and *Salmonella* Typhimurium, were tested using the disk diffusion method and MIC method. Ciprofloxacin was used as a control sample for antimicrobial activity measurement (Tables 3 and 4).

Our study is one of the first studies to examine the antimicrobial effect of this plant extract on these bacteria. Firstly, the antimicrobial effect of the essential oils of this plant *Ericamanipuliflora *grown in Syria was investigated by Tlas *et al. *(47). This study belonging to the authors showed that essential oil of *Erica manipuliflora* had moderate activity against the pathogenic bacteria, with minimum bactericidal concentration (MBC) values ranging from 8 to 32 mg mL^-1^. Since the antimicrobial effect was examined with the crude extract in our study, it can be accepted that it has an effect at high concentrations (312 mg mL^-1^).

When Table 3 was examined, it was determined that the 312 mg mL^-1^ plant extract created a 3 ± 0.28 mm inhibition zone diameter on *S. aureus* and Salmonella *Typhimurium*, while its inhibition effect on the *E. coli *was 4 ± 0.07 mm. The obtained inhibition effect for extract was found to be quite low compared to the ciprofloxacin. When the MIC results were examined, the most effective of 10 μL plant extract inoculum at 6 different concentrations (312 mg mL^-1^, 156 mg mL^-1^, 78 mg mL^-1^, 39 mg mL^-1^, 20 mg mL^-1^ and 10 mg mL^-1^) was found as 312 mg ml^-1^ (Table 4). The obtained results revealed that the substances found in the researched plant had slightly antimicrobial effects against the specified microorganisms. Similar results were also reported for *Erica *species by Guendouze-Bouchefa *et al., *(5), Turgay and Esen (37). Also, Kıvçak *et al., *(48) reported that all extracts of *Erica *showed antimicrobial activity against some bacteria but had no against the effect on observed yeast (*Candida albicans*). Similarly, the antibacterial effect of *Erica multiflora *was found weak against *Staphylococcus aureus *with a MIC (1000 mg L^-1^) method by Rios *et al.,* (49). Kacar *et al.* (50) investigated antimicrobial activity *Erica Manipuliflora* Salisb. which is endemic in Turkey (Muğla, Datça), against microfouling bacteria with using the MIC and Disc diffusion methods. At the end of their study, it was determined that their inhibition zones ranged between 8 and 15 mm. They had a strong antimicrobial activity. Upon the completion of the microdilution analysis, minimum values were determined as 19.5 mg mL^-1^. In another study, reported by Kıvçak *et al.* (48), the ethanol extracts of *Erica bocquetii* showed high antibacterial activity against some pathogens *(Salmonella* Typhimirium CCM 5445, *Staphylococus aureus* ATCC 6538P, *Escherichia coli* ATCC 29998).

The antimicrobial activity of the *Erica *family was attributed to phenolic compounds. Because polyphenols and tannins in this plant possess a strong binding ability to proteins or glycoproteins, also according to our knowledge, the antimicrobial activity of EMS could be associated with flavonoid aglycones, flavonoid glycosides, phenolic acids and derivatives, and flavan-3-ols and proanthocyanidins, the main components of EMS in our study, which is already known to exhibit antibacterial effects (37). These compounds may bind the bacterial adhesins and disturb the receptor exposition on the cell surface. Determination of many properties such as antimicrobial and antioxidant properties of EMS that grows naturally in our country would be advantageous in use in medicine, pharmacy, and industry. Some studies showed that the therapeutic effects of herbs were due to the synergistic effect of multiple compounds rather than a single active ingredient. In addition, it was determined that the active herbal substances provided a more effective treatment on antibiotic-resistant microorganisms (51).

**Table 1:** Quantitative determination of phenolic compound content of EMS by LC-MS/MS method

**Standard compounds**	^a^ **MRM**	^b^ **RSD (%)**	^c^ **LOD/LOQ** **(μg L** ^-1^ **)**	**Recovery (%)**	^d^ **RT**	^e^ **R** ^2^	**Equation**	**Concentration** **(µg L** ^-1^ **)**
Quercetin	301.1 > 151	0.0136	22.5/25.7	1.001	3.891	0.999	Y = (13.7831) X + (-146.951)	174.36
Acetohydroxamic Acid	76.10 > 43.10	0.0082	2.8/8.2	1.000	0.406	0.999	Y = (150.982) X + (23.1833)	84.37
Catechin hydrate	291.10 > 139.00	0.0236	8.2/11.4	0.994	2.532	0.999	Y = (79.2933) X + (-2406.22)	261.27
Vanillic Acid	168.80 > 93.00	0.0062	125.5/142.2	1.001	2.762	0.998	Y = (48.0522) X+ (-876.904)	520.01
Resveratrol	229.10 > 135.00	0.0131	9.0/13.6	0.998	3.606	0.998	Y = (46.4361) X + (-1314.61)	26.06
Fumaric Acid	115.20 > 71.00	0.0047	25.2/31.3	0.997	0.809	0.999	Y = (20.2986) X + (-762.592)	411.06
Gallic acid	169.20 > 125.00	0.0136	0.90/1.6	1.000	1.278	0.999	Y = (65.3835) X + (-2699.84)	14.87
Caffeic Acid	179.20 > 135.00	0.0137	6.3/10.7	1.009	2.836	0.996	Y = (124.785) X + (-487.132)	37.91
Phloridzin dihydrate	435.00 > 273.10	0.0564	61.0/207.0	1.00	3.594	0.999	Y = (33.4069) X + (-1396.90)	98.31
Oleuropein	539.10 > 377.20	0.0694	0.05/1.0	0.997	3.567	0.999	Y = (25.9240) X + (-558.916)	14.31
Ellagic Acid	300.90 > 145.10	0.0856	0.101/0.333	1.002	3.681	1.000	Y = (5.25903) X + (-1167.31)	N.D.
Myricetin	317.10 > 150.90	0.0079	55.4/59.6	0.999	3.644	0.999	Y = (37.0934) X + (2684.23)	131.18
Protocatechuic acid	181.20 > 108.00	0.0129	30.3/35.4	1.011	3.556	0.994	Y = (526.954) X + (23026.1)	N.D.
Butein	271.10 > 135.00	0.0145	22.7/28.6	0.096	3.935	0.999	Y = (49.3543) X + (367.917)	61.67
Naringenin	271.10 > 150.90	0.0205	5.4/6.4	0.998	3.952	0.996	Y = (317.241) X + (33733.3)	N.D.
Luteolin	285.20 > 132.90	0.0057	0.5/2.5	1.007	4.069	0.998	Y = (34.6668) X + (3721.79)	N.D.
Kaempferol	285.10 > 116.90	0.0144	206.6/214.3	0.999	4.298	0.999	Y = (2.63905) X + (-206.494)	8.83
Alizarin	239.20 > 210.90	0.0351	65.2/77.5	0.966	4.594	0.998	Y = (3.97487) X + (1614.23)	N.D.
4-Hydroxybenzoic Acid	137.20 > 93.00	0.0154	30.5/40.25	0.996	3.664	0.999	Y = (735.804) X + (-498.102)	N.D.
Salicylic acid	137.20 > 93.00	0.0124	4.2/7.6	1.009	3.558	0.999	Y = (746.369) X + (6072.41)	N.D.

^a^MRM: Multiple Reaction Monitoring; ^b^RSD: Relative standard deviation; ^c^LOD/LOQ (µg L^-1^): limit of detection/limit of quantitation; ^d^RT: Retention time; ^e^R^2^: Determination coefficient; N.D: Not detected.

**Table 2:** The radical scavenging, reducing power activity, and inhibition effect on AChE of EMS

**Samples**	**DPPH** ^a^ **(0.2 mg mL** ^-1^ **)**	**ABTS** ^a^ **(0.2 mg mL** ^-1^ **)**	**FRAP** ^b^ **(0.2 mg mL** ^-1^ **)**	**CUPRAC** ^b^ **(0.2 mg mL** ^-1^ **)**	**AChE**	
					**IC**_50_ ** (mg ml** ^-1^ **)**	**R** ^2^
EMS	18.31 ± 0.17	22.45 ± 0.43	0.251 ± 0.008	0.401 ± 0.009	0.124 ± 0.008	0.965 ± 0.002
BHA	71.82 ± 3.65	83.67 ± 2.62	0.454 ± 0.013			
BHT	46.33 ± 2.36	48.35 ± 1.99	0.624 ± 0.021			
Trolox	81.19 ± 6.63	80.06 ± 5.65	0.252 ± 0.009	0.516 ± 0.012		

Standard antioxidants (BHA, butylated hydroxyanisole; BHT, butylated hydroxytoluene, trolox).

^a^Values are expressed as percent radical scavenging activity.

^b^ Values are expressed as absorbance. High absorbance indicates high metal reduction capacity.

**Table 3:** Antibacterial effect of *Erica manipuliflora* Salib. extract (Inhibition zone, mm)

**Samples** **(6.24 mg/disk)**	**Concentration**	**Inhibition zone diameter (mm)** ***Staphylococcus aureus***	**Inhibition zone diameter (mm** ***)*** ***Esherichia coli***	**Inhibition zone diameter (mm)** ***Salmonella Typhimurium***
EMS	312 mg mL^-1^	3 ± 0.28	4 ± 0.07	3 ± 0.21
Control (Ciprofloxacin)	5 μg	19 ± 0.35	18 ± 0.14	16 ± 0.42

**Table 4:** MIC result of *Erica manipuliflora Salib*. extract (mg mL-1)

***Erica manipuliflora***	**Concentration** **(mg mL** ^-1^ **)**	**Inoculum amount (μL)**	***Staphylococcus aureus***	***Esherichia coli***	***Salmonella Typhimurium***
	312	10	-	-	-
	156	10	+	+	+
	78	10	+	+	+
	39	10	+	+	+
	19.5	10	+	+	+
	9.75	10	+	+	+
Medium+Inoculum	0	10	+	+	+
Medium+Solvent (DMSO)	0	10	+	+	+
Medium	0	0	-	-	-

(+): Growth, (-): No Growth

## Conclusion

In conclusion, the ethanol extracts of EMS have anticholinergic, antioxidant (metal reduction, radical and lipid peroxidation inhibition), and antimicrobial activities. It can be said that the chemo-diversity of plants has many bioactive properties due to its rich phenolic compound content. The medicinal EMS may be a potential candidate for the treatment of Alzheimer’s and many oxidative stress-related diseases due to its inhibitory effect on AChE and its antioxidant and antibacterial activity. Also, it can be considered as a promising source as a diet agent and for pharmaceutical or agro-food industries. Also, the finding results gave an idea about this plant that might be evaluated for various purposes, including its antioxidant, phenolic compound content, anticholinergic activity, linoleic acid peroxidation inhibitory activity, and antimicrobial properties.
